# Antibiotic treatment for ocular toxoplasmosis: a systematic review and meta-analysis: study protocol

**DOI:** 10.1186/s13643-019-1067-8

**Published:** 2019-06-20

**Authors:** John E. Feliciano-Alfonso, Andrés Vargas-Villanueva, María Alejandra Marín, Laura Triviño, Natalia Carvajal, Manuela Moreno, Tatiana Luna, Clara Lopez de Mesa, Juliana Muñoz-Ortiz, Alejandra de-la-Torre

**Affiliations:** 1 Departamento de Medicina Interna, Facultad de Medicina, Universidad Nacional de Colombia, Bogotá, Colombia; 2 Escuela de Medicina y Ciencias de la Salud, Universidad del Rosario, Bogotá, Colombia; 3 Escuela Superior de Oftalmología-Instituto Barraquer de América, Bogotá, Colombia; 4 NeURos research group, Escuela de Medicina y Ciencias de la Salud, Universidad del Rosario, Carrera 24 # 63 C 69, Bogotá, Colombia

**Keywords:** Toxoplasmosis, ocular, Toxoplasma, Uveitis, Anti-bacterial agents

## Abstract

**Background:**

Ocular toxoplasmosis (OT) is the most common cause of posterior uveitis, leading to visual impairment in a high proportion of patients. Antibiotics and corticosteroids lower the risk of permanent visual impairment by reducing the size of the retinochoroidal scar, the risk of recurrence, and the severity and duration of acute symptoms. Although OT is a very common cause of infectious posterior uveitis, its treatment remains controversial. Through our systematic review and meta-analysis, we aim to provide the best possible evidence-based information on the safety and effectiveness of the different antibiotic regimes for OT.

**Methods:**

This systematic review protocol has been developed based on PRISMA-P guidelines for reporting systematic reviews evaluating health care interventions. We will include all published and unpublished randomized controlled trials (RCTs) comparing different antibiotics used for the treatment of OT. We will consider changes in visual acuity, number of recurrences, improvement or worsening of ocular inflammation, size of lesion, and adverse effects as our outcomes. Screening, data extraction, and quality assessment will be undertaken by two reviewers with disagreements resolved through discussion. Studies that compared antibiotics with placebo will be excluded. The reviews will be assessed for quality and relevance. We will assess the risk of bias in five domains according to Cochrane group’s tool. The type of data will dictate measures of treatment effect. We will use a random-effects model to calculate our meta-analysis, as eligible studies represent clinically varied populations of participants.

**Discussion:**

The strength of our study will lie in the exhaustive and systematic nature of the literature search, as well as in its methods for assessing quality and analyzing RCT data. Considering the controversial efficacy of the treatment for OT, our study will contribute to improving the existing evidence on the effectiveness of different antibiotics. Future studies may be conducted to increase physicians’ awareness of antibiotic therapies, improving the health of patients with OT.

**Systematic review registration:**

PROSPERO CRD42018085468.

**Electronic supplementary material:**

The online version of this article (10.1186/s13643-019-1067-8) contains supplementary material, which is available to authorized users.

## Background

### Description of the condition

Ocular toxoplasmosis (OT) is the most common cause of posterior uveitis, and is the result of an acquired or congenital infection by the parasite Toxoplasma gondii (*T*. *gondii*). Congenitally acquired OT results from vertical transmission from mother to child, and may become apparent at birth or later, depending on the severity and location of the retinochoroidal lesions and if there is CNS compromise. Postnatally acquired OT becomes apparent when symptoms associated with an active retinochoroidal lesion are present. The sources of infection are food and water contaminated with oocysts from feline stool, or meat contaminated by tisular cysts. Clinical manifestations associated with the two etiologies are frequently indistinguishable. Positive IgG and IgM antibodies are found in patients with postnatally acquired OT if the infection is recent.

The most common manifestation of ocular toxoplasmosis is toxoplasmic retinochoroiditis (TR) which is typically a unilateral, unifocal, retinochoroidal lesion, usually associated with vitritis [1]. Even though the ocular signs of TR are highly suggestive of this disease, they may be mimicked by other infections [2]. Furthermore, in some cases, the symptoms may be atypical [3, 4], prompting the need to strengthen the evaluation by including biological diagnostic confirmation of OT [5]. Granulomatous anterior chamber inflammation is frequent, and retinal vasculitis (usually arteriolitis) is present in about a third of patients [1]. Visual acuity loss during acute TR results from vitritis or from involvement of the macula and the optic nerve. Visual loss may be permanent due to formation of a macular scar or optic atrophy. The scarring resulting from TR can be associated with severe visual field loss when it occurs in the macula or close to the optic disc [1].

The prevalence of TR follows the same pattern as general toxoplasmosis—varying greatly between regions of a country—and an estimated 25–30% of the world population is infected [6]. Low prevalences have been reported in southeastern Asia, North America, Northern Europe, and Sahelian countries of Africa (10–30%) [3, 4]. Moderate prevalences (30–50%) have been reported in Central and Southern Europe, and higher prevalences have been reported in Latin America and tropical African countries [7, 8].

OT in Europe and South America presents differently with respect to epidemiology, clinical manifestations, and immunology. Concerning epidemiology, OT is more common in South America, Central America, the Caribbean, and some parts of tropical Africa compared with Europe and North America, and it is very unusual in China. Ocular infection in South America is more severe than in other continents due to the existence of particularly virulent genotypes of the parasite [9–12] and the characteristics of the disease also differ in diverse areas of the world [13]. This variability yields significant consequences for therapeutic approaches [14] (higher macular involvement, vitreous and anterior chamber inflammation, bilateral involvement, strabismus, and synechiae) [5, 13].

Evaluation of cohorts of congenitally infected children showed that congenital toxoplasmosis was more frequently symptomatic in South America than in Europe (50–65% of the children developed ocular lesions) [15, 16]. In Colombia, a South American country, the lethality rate in congenitally infected children with lack of prenatal therapy can be as high as 25% [17].

Traditionally, antibiotics and corticosteroids have been the mainstay of pharmacologic therapy against *T*. *gondii.* Treatment is given to reduce the risk of permanent visual impairment (aiming to reduce the size of the retinochoroidal scar), the risk of recurrence, and the severity and duration of acute symptoms. Antibiotics are usually given for 6 to 8 weeks. Steroids are also sometimes used to decrease the severity of intraocular inflammation symptoms [18]. The aim of the treatment of gestational toxoplasmosis is to prevent fetal infection [19].

Antibiotics used for the treatment of TR have included trimethoprim-sulfamethoxazole, pyrimethamine, sulfadoxine, sulfadiazine, clindamycin, tetracyclines, clarithromycin, azithromycin, atovaquone, minocycline, spiramycin, rifabutin, trimetrexate, lincomycin, dapsone, sulfafurazole, ciprofloxacin, doxycycline, miokamycin, erythromycin, macrolide, sulfonamide, sulfamerazine, nifurtimox, methotrexate, alone or in combination [18, 20–23].

Several drugs are used in the treatment of toxoplasmosis. They act primarily against tachyzoites, and do not affect encysted forms. The synergistic action of pyrimethamine and sulfonamides has been demonstrated to interfere with parasitic replication by means of inhibiting its folate pathway. Spyramicin, a macrolide, can be used for the treatment of pregnant women because it has not been shown to be teratogenic [19]. However, in case of an established fetal infection (through ultrasonography amniocentesis), pyrimethamine and sulfadiazine plus folinic acid should be used after 18 weeks of gestation since pyrimethamine is potentially teratogenic [24]. Other macrolides, notably azithromycin, are also effective. Clindamycin, a Lincomycin, inhibits *T*. *gondii* by an unknown mechanism that involves the parasite organelle apicoplast. Other drugs against *T*. *gondii* include Dapsone, Azithromycin, Minocycline, and Rifabutin. Combinations of drugs are thought to be more effective [6, 19].

A key challenge is the development of a drug able to eliminate the cyst stage of the parasite, allowing it to effectively surpass host immuno-surveillance and drug pharmacodynamics. The ideal agent should be concentrated in the eye and should be able to effectively eliminate bradyzoites and tachyzoites as well as to penetrate cyst walls. It should also be well tolerated, causing no adverse effects [6].

### Objective of the study

Although OT is a very common cause of infectious posterior uveitis, its treatment remains controversial [6, 19–21, 25]. In many patients, *T*. *gondii* infection is asymptomatic and has been considered to need no treatment [6]. There are few studies comparing treatment regimes. In most patients, treatment needs to be continued for at least 4–6 weeks, increasing the likelihood of causing side effects [6]. Through our systematic review and meta-analysis, we aim to provide the best possible evidence-based information on the antibiotic treatment for OT.

Our primary objective is to determine the effects and safety of the different existing antibiotic treatment regimens for OT.

## Methods/design

This systematic review protocol was developed based on Preferred Reporting Items for Systematic Reviews and Meta-Analysis Protocols (PRISMA-P) guidelines for reporting systematic reviews evaluating health care interventions [26, 27].

### Criteria for considering studies in this systematic review

#### Types of studies

We will include all published and unpublished RCTs, comparing different antibiotics used for the treatment of our condition of interest. Studies which compared antibiotics with placebo will be excluded because a systematic review of this comparison has already been conducted by Pradhan et al. [18].

#### Types of participants

We will include studies involving patients of any age with OT who received antibiotic treatment for acute TR as well as those with healed scars who received prophylactic antibiotic treatment to prevent recurrent or new lesions, including immunocompetent patients, immunosuppressed patients, pregnant women, and children.

#### Types of interventions

We will include any type of antibiotic treatment regime known to be effective against *T*. *gondii* in any dosage, duration, and administration route that was compared against another antibiotic regime in any dosage, duration, and administration route. Therefore, we will include trimethoprim-sulfamethoxazole, pyrimethamine, sulfadoxine, sulfadiazine, clindamycin, tetracyclines, clarithromycin, azithromycin, atovaquone, minocycline, spiramycin, rifabutin, trimetrexate, lincomycin, dapsone, sulfafurazole, ciprofloxacin, doxycycline, miokamycin, erythromycin, macrolide, sulfonamide, sulfamerazine, nifurtimox, methotrexate, alone or in combination.

#### Types of outcome measures

As treatment is given to prevent long-term visual loss, our primary outcomes are:

- Change in visual acuity (VA) (using any measures), at least 3 months after the start of treatment: visual acuity refers to the ability of the visual system to resolve detail [28] and is expressed with the LogMAR (minimum angle resolution) scale which uses lines increasing in size by increments of 0.1 logarithmic units. LogMAR is a standardized optotype scale recommended for use by organizations such as the World Health Organization, the International Council of Ophthalmology, and the Royal College of Ophthalmologists for its accuracy and reliability in the measurement of VA. It is also well-correlated with other scales used for the measurement of VA. The MAR indicates the angular size of the smallest detail that an observer is able to identify in the optotype and is calculated by finding the inverse of the decimal value of the VA (MAR = 1/VA); however, in practice, the decimal logarithm of the MAR is often used (LogMAR as: logMAR = -log (VA)). Therefore, the maximum VA corresponds with 0 and the minimum VA with 1.0 [29, 30].
- Number of recurrences at the end of follow-up (any duration), defined as new onset of symptoms (decrease visual acuity, onset of blurred vision, floaters, hyperemia, photophobia, and pain) and/or signs (newly active lesions, anterior [aqueous humor cells] or posterior [retinochoroiditis with or without vitreous haze or cells] segment inflammation).

As the secondary aim of treatment is the reduction of severity and duration of pain and visual loss due to acute inflammation, our secondary outcomes are:

- Improvement or worsening of ocular inflammation signs according to the Standardization of Uveitis Nomenclature (SUN) Project measure, a standardized scale for grading the anatomic location and degree of activity of inflammation according to the presence of cells in the anterior chamber when examined with the slit lamp [31]. Improvement in the inflammation is defined as either a two-step decrease in the level of inflammation or a decrease to “inactive” (grade 0 cells), and worsening of the inflammation is defined either as a two-step increase in the level of inflammation or an increase to the maximum grade [32].
- Size of lesion at the end of follow-up (measured in optic disc diameters or micrometers).
- Adverse events (e.g., gastrointestinal symptoms, rashes and other allergic processes, decreased platelet or white blood cell count) (any mentioned).
- Duration of active lesion (number of weeks).

### Search methods for identification of studies

We will attempt to identify as many relevant RCTs as possible that investigate antibiotics for OT, with no restrictions regarding their publication language, date or status (published, unpublished, in press and in progress). We will perform an electronic database search, as well as manual searching, according to the Cochrane Handbook for Systematic Reviews of Interventions [33].

### Electronic searches

We will develop a highly sensitive systematic search in order to identify as many relevant RCTs as possible that investigate the efficacy of antibiotic treatment for ocular toxoplasmosis, irrespective of language, publication date, and publication status (published, unpublished, in press, and in progress). We will use both electronic searching in bibliographic databases and “handsearching,” as described in the Cochrane Handbook for Systematic Reviews of Interventions [33]. The results of all searches will be downloaded and managed using bibliographic software. Duplicate records of the same study will be deleted.

For this purpose, we will use a combination of exploded controlled vocabulary (MeSH, Emtree, DeCS) and free-text terms (considering spelling variants, plurals, synonyms, acronyms, and abbreviations) with field labels, truncation, proximity operators, and Boolean operators. The search strategies can be found in Additional file 1 (Electronic search strategies).

Specifically, we will conduct our search in the following electronic databases:

- MEDLINE, Ovid platform: inception to present.
- MEDLINE In-Process & Other Non-Indexed Citations, Ovid platform: inception to present.
- MEDLINE Daily Update, Ovid platform: inception to present.
- EMBASE.com: inception to present.
- The Cochrane Central Register of Controlled Trials (CENTRAL), Ovid platform: inception to present.
- LILACS, iAHx interface: inception to present.

When using the MEDLINE database, we will employ the highly sensitive Cochrane search strategy in order to identify RCTs [33]: sensitivity and precision maximizing version (2008 revision), Ovid format (Higgins 2011). The LILACS search strategy will be combined with RCT filters of the iAHx interface. These searches will be updated within 6 months of publication.

### Different research sources

For identification of additional studies, we will search these resources:

1. Clinical Trials Registries:
  WHO International Clinical Trials Registry Platform (ICTRP) portal (http://apps.who.int/trialsearch/): inception to present.
  ClinicalTrials.gov (http://clinicaltrials.gov/): inception to present.
2. Search for gray literature at the System for Information on Gray Literature in Europe “OpenGrey” (http://www.opengrey.eu/): inception to present.
3. Manual search within reference lists for all relevant studies identified by other methods.

### Data collection and analysis

#### Selection of studies

For studies obtained throughout the electronic search, two review authors (John Feliciano-Alfonso and Andrés Vargas) will independently review titles and abstracts of all studies and retrieve potentially relevant studies in pdf format. They will also review the pdf files against the inclusion criteria. Disagreements will be resolved by consensus or by independent evaluation by a third author (Alejandra de-la-Torre).

### Data extraction and management

A data recollection form will be designed. Two review authors (AV, JFA) will independently extract relevant details about the design and the results of each study. Disagreements will be resolved by consensus or by independent evaluation by a third author (ADLT). Those authors will be area and methodology experts. The extracted information will include:

1. Study year and author
2. Study location and setting
3. Study design
4. Power calculation performed
5. Inclusion criteria
6. Exclusion criteria
7. Basal participant information and features
8. Total number of intervention groups
9. Types of interventions
10. Types of comparison
11. Antibiotics used (dose, frequency, route of administration)
12. Randomization methods
13. Allocation concealment methods
14. Number of included, randomized and analyzed subjects
15. Masking method
16. Number of participants lost during follow-up
17. Differences in the outcome assessment between groups
18. Time of participant follow-up to measure outcomes
19. Adverse effect reports validation method
20. Whether intention-to-treat analysis was performed or not
21. Funding resources
22. Ethical aspects (ethics committee approval, informed consent signing)

JFA will enter data into Review Manager (RevMan) and a third reviewer (CL) will check them to ensure data quality. When information regarding any of the above is unclear, we will contact the authors of the studies in order to ask for further details.

### Assessment of risk of bias in included studies

We will use the Cochrane Collaboration’s “Risk of bias” tool for RCTs and criteria in the Cochrane Handbook for Systematic Reviews of Interventions to assess these in the relevant domains of the reported methods and results [32].

### Measures of treatment effect

#### Dichotomous data

For dichotomous data, we will present the results as a risk ratio (RR) with 95% confidence intervals (CI). The RR will be used as a relative effect measure which works well with a low or high rate of events and is easy to interpret and use in clinical practice.

#### Continuous data

For continuous data, we will use the mean difference if outcomes are measured in the same way between trials. We will use the standardized mean difference to combine trials that measure the same outcome, but use different methods. All effect estimates will be presented with 95% confidence intervals.

#### Dealing with missing data

We will contact the study investigators in order to obtain the missing data. For included studies, we will note levels of attrition. We will address the potential impact of missing data on the findings of the review in the “Discussion” section. We will analyze only the available data without making assumptions or imputing data.

#### Assessment of heterogeneity

We will assess statistical heterogeneity in each meta-analysis using the *I*^2^ statistic. We will consider heterogeneity as substantial if *I*^2^ is greater than 40% and less than 70%, which would require the use of random effects model in the analysis. If there is substantial heterogeneity and *I*^2^ is greater than 70%, meta-analysis will not be carried out and a narrative synthesis will be conducted instead.

#### Assessment of reporting biases

We will use funnel plots to assess publication bias when at least ten studies are available for meta-analysis. We will evaluate possible sources of asymmetry in funnel plots according to Chapter 10 of the *Cochrane Handbook for Systematic Reviews of Interventions* [32].

### Data synthesis

We will use a random effects model to calculate our meta-analysis if eligible studies represent clinically varied populations. We will use fixed effect meta-analysis for combining data where it is reasonable to assume that studies are estimating the same underlying treatment effect. We will carry out statistical analysis using RevMan.

### Sensitivity analysis

We will conduct a sensitivity analysis by excluding studies at high risk of bias.

### Summary of findings table

We will use the Grading of Recommendations Assessment, Development and Evaluation (GRADE) approach in order to produce a summary of findings table. We will downgrade the quality of evidence depending on the presence of the following factors:

1. Risk of bias
2. Inconsistency of results
3. Indirectness of evidence
4. Imprecision
5. Publication bias

The quality level will be upgraded for:

1. Large effect
2. Plausible residual confounding
3. Dose response gradient

We will include the following outcomes in the “summary of findings” table:

1. Visual acuity
2. Recurrence of retinochoroiditis
3. Intraocular inflammation
4. Size of lesion
5. Adverse effects

## Discussion

This systematic review and meta-analysis will provide efficient and robust information on the state-of-the-art evidence for the treatment of OT.

The strength of this systematic review and meta-analysis will lie in the exhaustive and systematic nature of the literature search, in its methods for assessing quality and analyzing RCT data, including consideration of the sample size and methodological flaws of included studies.

Our study will contribute to improving the existing evidence on the effectiveness of the different antibiotic regimes for the treatment of OT. Future studies may be conducted to increase physicians’ awareness of antibiotic therapies, improving the health of patients with OT.

Furthermore, it will help to identify inconsistent evidence so further research can be done accounting for previous poor-quality trials.

## Additional file

Additional file 1: Electronic search strategies. (DOCX 19 kb)

## Abbreviations

ADLT: Alejandra de la Torre
AV: Andrés Vargas
CI: Confidence intervals
GRADES: Grading of recommendations assessment, development and evaluation
JFA: John Feliciano
OT: Ocular toxoplasmosis
PRISMA P: Preferred Reporting Items for Systematic Reviews and Meta-Analysis Protocols
RevMan: Review manager
RR: Risk ratio
SUN: Standardization of uveitis nomenclature
TR: Toxoplasmic retinochoroiditis
VA: Visual acuity
