# Supplementary material for: Antibiotic treatment for ocular toxoplasmosis: a systematic review and meta-analysis: study protocol
Source: Syst Rev. 2019 Jun 20;8:146. doi: 10.1186/s13643-019-1067-8 (PMC6587239; doi:10.1186/s13643-019-1067-8)
Supplement: Supplementary file 1 — Electronic search strategies. (DOCX 19 kb) [file 13643_2019_1067_MOESM1_ESM.docx]

Additional file 1 (Electronic search strategies).

**MEDLINE and CENTRAL:**

1 exp Toxoplasmosis, Ocular/

2 exp Toxoplasma/

3 exp Toxoplasmosis/

4 toxoplasm$.tw.

5 gondi$.tw.

6 exp Chorioretinitis/

7 exp Retinitis/

8 exp Choroiditis/

9 exp Uveitis/

10 retinochoroiditis.tw.

11 chorioretin$.tw.

12 uveitis.tw.

13 ocular.tw.

14 retinitis.tw.

15 choroidoretinitis.tw.

16 choroiditis.tw.

17 eye.tw.

18 ophthalmic.tw.

19 or/2-5

20 or/6-18

21 19 and 20

22 1 or 21

23 exp Anti-Infective Agents/

24 exp Trimethoprim, Sulfamethoxazole Combination/

25 tmp-smx.tw.

26 (tmp adj5 smx).tw.

27 cotrimoxazole.tw.

28 trimethoprim$.tw.

29 exp Pyrimethamine/

30 pyrimethamine.tw.

31 exp Sulfadoxine/

32 sulfadoxine.tw.

33 exp Sulfadiazine/

34 sulfadiazine.tw.

35 exp Clindamycin/

36 clindamycin.tw.

37 exp Tetracyclines/

38 tetracycline$.tw.

39 exp Minocycline/

40 minocycline.tw.

41 exp Clarithromycin/

42 clarithromycin.tw.

43 exp Azithromycin/

44 azithromycin.tw.

45 exp Atovaquone/

46 atovaquone.tw.

47 exp Spiramycin/

48 spiramycin.tw.

49 exp Rifabutin/

50 rifabutin.tw.

51 exp Trimetrexate/

52 trimetrexate.tw.

53 exp Lincomycin/

54 lincomycin.tw.

55 exp Dapsone/

56 dapsone.tw.

57 exp Sulfisoxazole/

58 sulfafurazole.tw.

59 sulfisoxazole.tw.

60 exp Ciprofloxacin/

61 ciprofloxacin.tw.

62 exp Doxycycline/

63 doxycycline.tw.

64 exp Miocamycin/

65 mio?amycin.tw.

66 exp Erythromycin/

67 erythromycin.tw.

68 exp Macrolides/

69 macrolide$.tw.

70 exp Sulfonamides/

71 sulfonamide$.tw.

72 exp Sulfamerazine/

73 sulfamerazine.tw.

74 exp Nifurtimox/

75 nifurtimox.tw.

76 exp Methotrexate/

77 methotrexate.tw.

78 or/23-77

79 randomized controlled trial.pt.

80 controlled clinical trial.pt.

81 random$.ab.

82 placebo.ab.

83 clinical trials as topic.sh.

84 randomly.ab.

85 trial.ti.

86 or/79-85

87 exp animals/ not humans.sh.

88 86 not 87

89 22 and 78 and 88

CENTRAL strategy does not include 79 to 89 lines

**EMBASE**

1 'ocular toxoplasmosis'/exp

2 'Toxoplasma'/exp

3 'toxoplasmosis'/exp

4 toxoplasm*:ab,ti

5 gondi*:ab,ti

6 'chorioretinitis'/exp

7 'retinitis'/exp

8 'choroiditis'/exp

9 'uveitis'/exp

10 retinochoroiditis:ab,ti

11 chorioretin*:ab,ti

12 uveitis:ab,ti

13 ocular:ab,ti

14 retinitis:ab,ti

15 choroidoretinitis:ab,ti

16 choroiditis:ab,ti

17 eye:ab,ti

18 ophthalmic:ab,ti

19 or/2-5

20 or/6-18

21 19 and 20

22 1 or 21

23 'antiinfective agent'/exp

24 'cotrimoxazole'/exp

25 tmp-smx:ab,ti

26 (tmp NEAR/5 smx):ab,ti

27 cotrimoxazole:ab,ti

28 trimethoprim*:ab,ti

29 'pyrimethamine'/exp

30 pyrimethamine:ab,ti

31 'sulfadoxine'/exp

32 sulfadoxine:ab,ti

33 'sulfadiazine'/exp

34 sulfadiazine:ab,ti

35 'clindamycin'/exp

36 clindamycin:ab,ti

37 'tetracycline derivative'/exp

38 tetracycline*:ab,ti

39 'minocycline'/exp

40 minocycline:ab,ti

41 'clarithromycin'/exp

42 clarithromycin:ab,ti

43 'azithromycin'/exp

44 azithromycin:ab,ti

45 'atovaquone'/exp

46 atovaquone:ab,ti

47 'spiramycin'/exp

48 spiramycin:ab,ti

49 'rifabutin'/exp

50 rifabutin:ab,ti

51 'trimetrexate'/exp

52 trimetrexate:ab,ti

53 'lincomycin'/exp

54 lincomycin:ab,ti

55 'dapsone'/exp

56 dapsone:ab,ti

57 'sulfafurazole'/exp

58 sulfafurazole:ab,ti

59 sulfisoxazole:ab,ti

60 'ciprofloxacin'/exp

61 ciprofloxacin:ab,ti

62 'doxycycline'/exp

63 doxycycline:ab,ti

64 'miokamycin'/exp

65 mio?amycin:ab,ti

66 'erythromycin'/exp

67 erythromycin:ab,ti

68 'macrolide'/exp

69 macrolide*:ab,ti

70 'sulfonamide'/exp

71 sulfonamide*:ab,ti

72 'sulfamerazine'/exp

73 sulfamerazine:ab,ti

74 'nifurtimox'/exp

75 nifurtimox:ab,ti

76 'methotrexate'/exp

77 methotrexate:ab,ti

78 or/23-77

79 'randomized controlled trial'/exp

80 'single blind procedure'/exp

81 'double blind procedure'/exp

82 'crossover procedure'/exp

83 or/79-83

84 random*:ab,ti

85 placebo*:ab,ti

86 allocat*:ab,ti

87 factorial*:ab,ti

88 crossover*:ab,ti

89 'cross over':ab,ti

90 trial:ti

91 (doubl* NEXT/1 blind*):ab,ti

92 or/84-91

93 83 or 92

94 'animal'/de

95 'animal experiment'/de

96 'nonhuman'/de

97 or/94-96

98 'human'/de

99 97 and 98

100 97 not 99

101 93 not 100

102 22 and 78 and 101

**LILACS**

(mh:("Toxoplasmosis, Ocular")) OR (ti:(Toxoplasmosis)) OR (ab:(Toxoplasmosis)) AND db:("LILACS") AND type_of_study:("clinical_trials")

**WHO International Clinical Trials Registry Platform (ICTRP) portal**

Ocular toxoplasmosis

**ClinicalTrials.gov**

Ocular toxoplasmosis

**OpenGrey**

Ocular toxoplasmosis
